# Investigating the association between population density and travel patterns in Indian cities—An analysis of 2011 census data

**DOI:** 10.1016/j.cities.2020.102656

**Published:** 2020-05

**Authors:** Rahul Goel, Dinesh Mohan

**Affiliations:** a MRC Epidemiology Unit, University of Cambridge, UK; b Transportation Research and Injury Prevention Programme, Indian Institute of Technology Delhi, Hauz Khas, Delhi, India

## Abstract

Many transport planners consider urban population density to be a significant determinant of travel behaviour. Much of the evidence for this comes from research in low-density, high-income settings. The 2011 Census of India reported mode of travel to work and distance for the first time. We have used these data to investigate the effect of urban density on commute travel patterns at city-level for Indian cities. In addition, we investigated the relationship between travel behaviour and other city-level variables. Using regression, we found almost no independent effect of density on the mode share of walk, cycle, motorised two-wheelers, cars and public transport, after controlling for population and income levels for the cities. Further, it appears that once density levels are greater than ~80 persons per hectare (pph), other factors become more important in determining travel patterns in cities. This evidence has significant implications for urban planning and transport policy in Indian cities and for many other low- and middle-income cities where average density tends to be higher than ~80 pph. For these cities, growth in the use of sustainable transport may not depend on further densification of already dense cities, but on details of how neighbourhoods and streets are designed.

## Highlights

• Census in India reported travel mode and distance of work trips for the first time.
• Most Indian cities have higher density than the densest cities in the high-income countries.
• Large changes in population density have only negligent effect on travel patterns.
• Population size and city-level income are more important determinants of travel behavior.

## 1 Introduction

The number of cities in India with population >1 million increased from 35 to 53 between 2001 and 2011 (Census-India, 2012). Out of the total urban population living in about 8000 towns in India, >40% live in the 53 largest cities. The Census of India defines a town as a place that satisfies following three criteria—a minimum population of 5000; at least 75% of the male working population engaged in non-agricultural pursuits; and a population density of at least 400 persons per square kilometre (km^2^). In the past 20 years, the area of urban cover in the top 100 Indian cities has increased about 2.5 times (Nagendra, Sudhira, Katti, Tengö, & Schewenius, 2014). During the same period, vehicle ownership grew by 7 times at a compound annual rate of 10% (Guttikunda & Mohan, 2014).

The growth in population, average income levels of city dwellers, and number of large cities, coupled with an exponential increase of the vehicular fleet, is leading to increases in the consumption of fossil fuels (Goel, Mohan, Guttikunda, & Tiwari, 2016), emissions of greenhouse gases (GHG) (Singh et al., 2008), deterioration of urban air quality (CPCB, 2010; Guttikunda, Goel, & Pant, 2014), and a consistent growth in road traffic fatalities (Mohan, Tiwari, & Bhalla, 2013). These developments have resulted in the Government of India recognizing transport (including freight) as one of the major sectors contributing to GHG emissions in India and an important sector where policies are needed to control these externalities of transport (Planning Commission, 2011). There is an urgent need to understand the patterns of mobility, so that evidence-based countermeasures can be put in place.

Various reports addressing the externalities of transport have studied the linkage of the built-environment with travel behaviour. Built environment includes, and is not limited to, land-use mix, population density, street design features, and accessibility of public transport (Cervero, 2003; Ewing & Cervero, 2001). These effects on travel behaviour have been studied at three different spatial scales— metropolitan, intra-metropolitan, and local/neighbourhood (Zegras, 2010). The underlying objective of these studies has been to identify those modifiable factors that can help reduce vehicle kilometres travelled (VKT) by individuals for their daily needs. The reduction in VKT can be achieved through higher number of trips by walking, cycling or using public transport modes, reduction of travel distance, or reduction of number of trips, or a combination of all these.

Among the various built environment factors, population density has been studied the most (Salon, Boarnet, Handy, Spears, & Tal, 2012) and, as a result, has been given most relevance in terms of the policy implications (Mindali, Raveh, & Salomon, 2004). There is enough evidence to show that the built environment, in terms of the density and the land-use mix, affects travel behaviour such as mode choice as well as trip distance (Frank & Pivo, 1994; Cervero & Radisch, 1996; Ewing & Cervero, 2001; Leck, 2006). The evidence and growing belief in the relationship between the urban density and the travel demand resulted in an interest in the ‘compact cities’ among the policy makers (Breheny, 1995). Transit oriented development (TOD) is a classic example in which densification lies at the heart of the public transport friendly policy (Carlton, 2007; Thorne-Lyman et al., 2011). Increase in the density has been hypothesized to reduce motor vehicle use for many reasons. Higher densities are expected to result in the reduction of trip lengths facilitated by mixed land-use, encouragement of non-motorised travel, and to induce mode shift from private to public transport modes (Cervero, 2003). In other words, people are expected to walk to their destinations when they are close by (mixed-use), the trip lengths are likely to be low for a large fraction of population if they all live close to their destinations in a high density neighbourhood, number of people living close to public transport stops increases which leads to higher use.

In the context of urban density and its effects on travel behaviour, using official data from cities around the world, Newman and Kenworthy, 1989, Newman and Kenworthy, 1999 found a strong negative correlation between overall city population density and per capita gasoline use. The trends reported by the authors show an exponential reduction in the per capita fuel consumption as densities increased from their levels in American and Australian cities to those in Asia. Their evidence, along with those reported by other authors have had a significant influence on policy makers promoting higher density as an important means to reduce transport energy consumption (Mees, 2010; Mindali et al., 2004).

Recently, India has also witnessed land-use policy implications based on density. In a report on urbanisation in India, World Bank highlighted the need to increase the Floor Area Ratio (FAR) of metropolitan cities of India to enable densification. Interestingly, the recommendation was made solely on the basis of differences in the FAR policy of Indian cities and that of various western counterparts, while no attempt was made to compare the density expressed as persons per unit area (World Bank, 2016; Patel, 2013). Government of India has also taken initiatives to densify cities in lieu of TOD. For instance, some cities such as Indore and Ahmedabad have increased FAR along certain corridors to boost PT demand (MHUPA, 2016).

Criticism of such policies has revolved around the issue of whether to interpret density as a correlating variable or a causal factor affecting travel behaviour (Ewing & Cervero, 2001; Handy, 1995; Miller & Ibrahim, 1998; Steiner, 1994). It is hypothetised that the effect of density on travel may be due to other factors (Salon et al., 2012). There has been additional criticism of data reported by Newman and Kenworthy regarding the choice of cities and the neglect of variations among cities with similar densities (Mees, 2010; Mindali et al., 2004; Mohan, 2013). It has been argued that the densities below a critical level reduce the usage of public transport and non-motorised modes, but above a certain level the density may become less of a determining factor (Mohan, 2013). For example, according to a study conducted in Mumbai (Shirgaokar, 2016), the largest and one of the densest cities in India, while private vehicle use decreases with increasing population density, the effect size is so small, that even with 10% increase in density, there is <1% decrease in private vehicle use. Even a study from the US at much lower levels of density (Hong, 2017) concluded that beyond a certain level, density has no effect on transport emissions.

Studies have reported many other factors that impact travel behaviour. Metropolitan size (in terms of population) has been found to be associated positively with commuting distance. As cities become larger, there are many more varieties of specialised services and goods, and therefore, individuals are likely to travel longer in order to access jobs suited to their abilities. As cities grow in population they also become prosperous. With more prosperity, individuals are more likely to afford expensive modes of transport, and to live farther from their work location in order to live in larger homes (Stead & Marshall, 2001; Schwanen, 2002; Engelfriet & Koomen, 2018).

Most studies regarding travel behaviour and its linkages with the built environment have been based in the high-income countries, particularly in the USA (Ewing & Cervero, 2001; Leck, 2006). Most of these studies investigated how travel demand is impacted by suburbanization (or urban sprawl) and high vehicle ownership — a combination of characteristics that set American cities apart from their counterparts in North America and Europe (Pucher & Lefèvre, 1996). One needs to be careful before translating the conclusions from high-income countries to low-income settings such as India as there are no low-density cities in India (Guttikunda et al., 2014). In addition, even in high income settings, there is a great deal of variation in travel behaviour between cities with similar densities. This indicated that travel behaviour both in high income and low and middle-income countries may depend on many other factors than population densities.

Moreover, current car ownership in India is 15 per 1000 persons (MoRTH, 2012a, MoRTH, 2012b), while those in European countries is 400 to 600, and that in the North America is up to 800 (Huo & Wang, 2012). With such high access to private vehicles in high-income countries, individuals have higher independence in terms of deciding how far to go and when. In Indian cities, on the other hand, a large proportion of the population lacks vehicle ownership, and therefore, relies on walking, cycling, or public transport. Compared to a private car, non-motorised modes limit the distance of travel and public transport limits the spatial coverage as well as the distance.

In India, such research has been difficult due to the lack of publicly available data, such as national-level or city-level household travel surveys or census-based travel to work data. Travel surveys have been conducted in many cities in India to prepare urban mobility plans, (Wilbur Smith Associates, 2008). However, details of such data are not publicly available for researchers and the quality of such surveys has also been criticised (Mohan, 2013). The 2011 Census of India, for the first time, reported data on the mode of travel and distance for work trips for all districts in India.

In this paper we aim to achieve the following objectives:

a) To develop a database of Indian cities with harmonised set of variables on built environment, demographic and socioeconomic characteristics, and travel behaviour
b) To study the association of travel behaviour and population density at the city level, and to investigate the mediating effect of other city-level correlates
c) To put the relationship between density and travel behaviour in Indian cities within an international context

## 2 Data and method

### 2.1 Selection of cities

The 2011 Census of India reported mode of travel to work and travel distance (Census-India, 2016). These data were reported for the category of ‘other workers’, which is defined as the workers other than those involved in cultivation, agriculture labour, or a household–based industry. This is the first time that the details of travel-related data have been reported by the Census in India. These data, at the time of the current study, have only been reported at the district level, categorized as total, urban, and rural. A district is an administrative division within a state and, according to 2011 Census, there were 640 districts in India. A district consists of one or more towns and villages, and therefore, urban data at the district level will be the sum total of all the towns and cities in that district. In 2011, there were >8000 towns, out of which 475 are urban agglomerations (UA) with a population of 100,000 or more. The Census defines UA as a continuous urban spread constituting a town and its adjoining outgrowths, or two or more physically contiguous towns together with or without outgrowths.

In this paper, our objective is to understand travel to work and its various correlates at the city level. In the context of the urban travel patterns, it is important to analyse data at the city level since a city is largely a contiguous land mass, while a district on the other hand consists of fragmented urban areas. As a result, parameters such as the population of a district or its size are not relatable to a small town within it. To circumvent this problem, we made some assumptions. To approximate city-level data from that of the whole of district, we selected those UA that contribute at least 75% of urban population to their corresponding districts. This ensures that the urban data at district level is representative of the selected cities. Some UA include cities of more than one district. For those cases, we used the combination of all the constituting districts to calculate that data of district corresponding to those UA. A total of 72 UA were selected out of which 62 contributed >80% of the population to their corresponding district. In the subsequent text, the selected UA will be referred to as cities.

### 2.2 Population density and GDP

We estimated population density of the cities using district-level urban built-up area reported by the National Remote Sensing Centre through their web portal, Bhuvan (NRSC, 2016). The NRSC reports Land use Land Cover data using multi-temporal satellite data of 2011–12 from Resourcesat-2 LISS III images. We obtained corresponding district-level urban population from 2011 Census database, and expressed density as persons per hectare (100 ha = 1 km^2^). For our analysis, we assumed that the density of urban area estimated at the district level represents density at the city level. As a result, for some of the cities, density may be underestimated since it includes all the urban areas within the district.

We calculated size of the city, in terms of the area in km^2^, using district-level urban population density and city-level population. For GDP, we used district-specific data reported for year 2004–05 from the web portal of Open Government Data Platform India (OGD, 2014), since district-level GDP data for all the states was not available for 2010–11, the year corresponding to the census. We used the state-specific ratios of GDP of 2010–2011 to that of 2004–05 (Planning Commission, 2014) to estimate district-level GDP for 2010–11. We estimated GDP per capita using the total population (urban and rural) of the corresponding district. For five districts, GDP estimates were not available for either of the financial years. As a result, we report GDP data for 66 of the total 72 cities. We have expressed GDP in Indian Rupees (INR), unless otherwise mentioned.^1^

### 2.3 Vehicle ownership and mode share of work trips

In Census 2011, ownership of a vehicle type by a household is reported if the household owns at least one of the three vehicle types—cycle, MTW and car. The households have been classified among four categories based on their vehicle ownership, three for each of the vehicle types and one for none of the vehicles. The Census does not report the number of vehicles at the household level. The sum of the four categories for a given district may be >100% as it double counts the households owning more than one vehicle type. For this analysis, we did not include the last category of none of the vehicles.

Mode share has been classified among nine different categories: (1) walk, (2) cycle, (3) moped/scooter/motorcycle, (4) car, (5) tempo/autorickshaw/taxi, (6) bus, (7) train, (8) water transport, and (9) any other. We refer to Category 3 as motorised two wheelers (MTW), and category 5, which consists of intermediate public transport, or para-transit modes, common in most Indian cities (for their description see Goel & Tiwari, 2016), as IPT. A combination of categories 5, 6 and 7, consisting of different forms of public transport will be referred to as PT. We did not consider categories 8 and 9 for the current analysis, which contribute fewer than 1% of all work trips in India. Census reported mode-specific distance of trips categories into seven categories: 0–1 km, 2–5 km, 6–10 km, 11–20 km, 21–30 km, 30–50 km, and ≥ 50 km. For this analysis, we used distance distribution for a combined total of 9 travel-mode categories.

### 2.4 Regression

The measures of interest in this analysis are in the form of proportions such as mode shares, and share of trips in various distance bands. For these, we developed beta regression models. Beta distribution is defined on the interval 0 to 1 and is therefore appropriate to model proportions (Ferrari & Cribari-Neto, 2004). We used ‘betareg’ package in R to develop these regression models (Cribari-Neto & Zeileis, 2009).

## 3 Results

### 3.1 Descriptive statistics

**Fig. 1:**
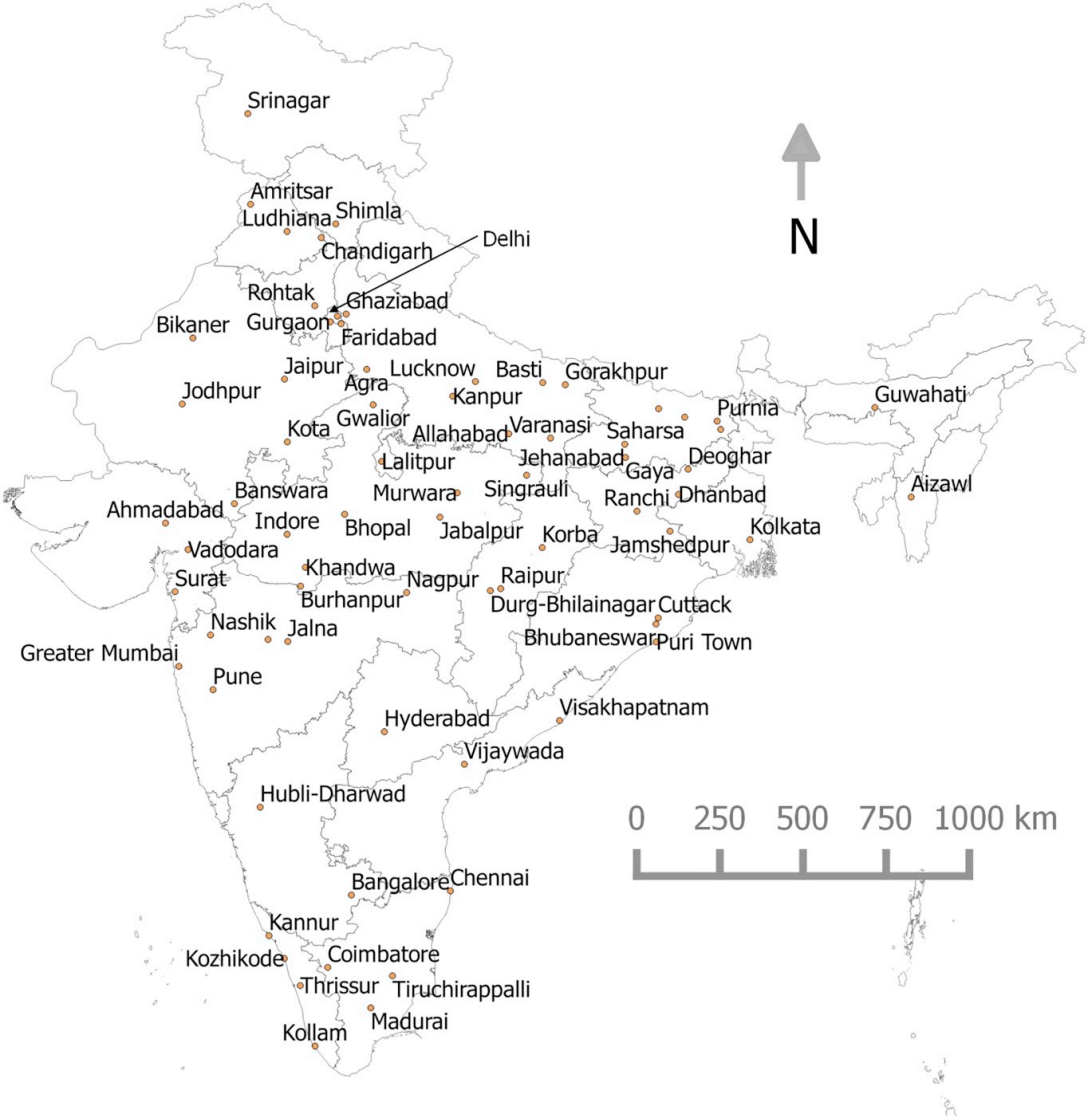
Case-study cities with state boundaries in India.

Table 1 presents descriptive statistics of the city-level variables considered in our analysis. The table also includes natural log of population. Fig. 1 presents a map of the 72 cities and city-specific data has been provided in Supplementary material. Average population of the cities is 2.2 million and range from 100,000 to 18.4 million. Three cities have population >10 million, five between 5 and 10 million, and thirty-six between 1 and 5 million. Thus, India has 3 of the 35 cities in the world with >10 million population (Demographia, 2015). Average GDP per capita of the cities is INR 68,000 per capita (At 2015 exchange rates US$ 1 = INR 65). According to the World Bank, GDP per capita for overall India was US$ 1456 in 2011, while that of the US was US$ 49,780, UK was US$ 41,020, and China was US$ 5570 (World Bank, 2016). Thus, India's per capita income is an order of magnitude lower than that of its western counterparts.

**Table 1:** Descriptive statistics of 72 cities.

Variable	Average	Median	Std. Deviation	Minimum	Maximum
Population (District)	4,442,524	3,054,640	5,514,163	400,309	38,205,210
GDP (in 10 million INR)	34,158	16,033	59,310	1523	351,264
GDP per capita (1000 INR)	68	57	50	14	315
Population Density (persons per hectare)	137	113	75	46	435
Population (City)	2,182,372	1,119,824	3,493,040	100,128	18,414,288
Ln (Population City)	13.8	13.9	1.2	11.5	16.7
Size (km^2^)	145	101	167	3	727

Vehicle ownership					
Car	8%	7%	6%	1%	30%
MTW	28%	29%	13%	5%	50%
Cycle	44%	44%	18%	2%	81%

Mode share					
Walk	30%	29%	9%	16%	57%
Cycle	20%	21%	10%	1%	38%
MTW	24%	25%	9%	2%	41%
Car	5%	4%	4%	1%	28%
IPT	5%	5%	3%	0%	19%
Bus	12%	7%	12%	1%	48%
Train	3%	2%	4%	0%	33%

Distance categories (all travel modes)					
0–1 km	22%	21%	6%	15%	48%
2–5 km	39%	39%	6%	24%	53%
6–10 km	21%	22%	5%	8%	32%
11–20 km	8%	7%	4%	2%	18%
21–30 km	5%	4%	2%	1%	12%
31–50 km	2%	2%	1%	1%	9%
> 51 km	2%	2%	1%	0%	10%

Average population density of the cities is 137 pph; twenty of the 72 cities (28%) have density higher than 150 pph, 27 (38%) between 100 and 150 pph, 17 (24%) between 70 and 100 pph, 7 between 50 and 70 pph and only 2 below 50 pph. All the 45 cities with population >1 million have density >90 pph with an exception of 3 cities, and the average density of the 10 largest cities is 200 pph. Thus, almost all Indian cities have higher density than the densest cities in the west. For instance, in year 2000, London (population: 6.1 million) had a density of 54 pph, Paris (9.6 million) had 65 pph, and New York (19 million) had 26 pph (Angel, Parent, Civco, & Blei, 2010).

On an average, less than one-tenth of all the households own at least one car, <30% own an MTW, and >40% own a cycle. In terms of the commute patterns, half of all the trips are either walked or cycled, one-fifth of all the trips use some form of public transport—IPT, bus, or train, a quarter of all the trips use MTW, and 5% trips use cars. >60% of all the trips are shorter than 5 km, and less than one-third of all trips are longer than 10 km. Among the western settings, 86% of the work trips in the US (2009; USCB, 2011), 64% in the UK (2011; Gower, 2013) and 62% in the Netherlands (2007; MOT, 2009) were carried out using cars.

### 3.2 Correlations between variables

Given that there are multiple variables corresponding to each city, it is important to understand the interrelationships between the variables. For this, we calculated the Pearson correlation between different variables, as presented in Table 2 along with their respective significance levels. City population is positively correlated with population density, GDP per capita, ownership of car and MTW, size of the city, share of motorised modes of transport (car, bus, train), and share of trips longer than 5 km. Compared to the absolute value of population, its natural log has a much higher correlation with most variables, indicating a non-linear relationship. In conclusion, as the cities become larger, they tend to be denser, richer, and the workers commute longer and are more likely to use motorised private and public transport modes.

**Table 2:**
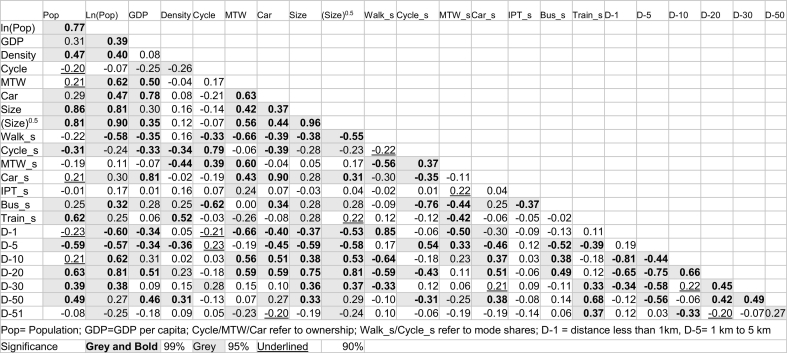
Pearson correlation between the city parameters.

GDP per capita also has a positive correlation with all the variables population is correlated with. More importantly, GDP per capita shows high correlation with the car ownership (0.78) and its mode share (0.81), mode share of bus, and with distance categories 11–20 km and 30–50 km. Higher GDP is also associated with lower share of walking and cycling in commuting. Density has a positive correlation with car ownership, mode share of PT, and trips longer than 10 km. More significantly, density is negatively correlated with the ownership of cycle and its mode share, mode share of MTW, and distance category 2–5 km. The positive association between longer distance commute and population density is counterintuitive, however, this is probably because higher density is linked with larger city size.

There is correlation between mode shares of different vehicle types with the specific distance categories. Walking has the highest positive correlation (0.85) with distance <1 km, cycling and MTW with distance of 2–5 km (0.54 and 0.33, respectively), car and bus with distance category of 11–20 km (0.51 and 0.49, respectively), and train with distance category 30–50 km (0.68). This highlights a strong link between type of travel modes and travel distance, with walking catering to the shortest trips, cycling and MTW to the mid-length trips, car and PT to the long trips, and train to the longest of the trips. This is also the reason that the bus mode share is negatively correlated with that of the cycle (−0.76), MTW (−0.44) and IPT share (−0.37), and positively related to car mode share (0.25). This indicates that as cities become larger in population, or have longer commute distance, the two are highly correlated as discussed above, travel modes catering to the longer travel distance attain a higher share. Among public modes, the share of IPT reduces, and that of the bus increases, and among private modes, share of cycle and MTW reduces and that of the car increases.

### 3.3 Population density and population size

**Fig. 2:**
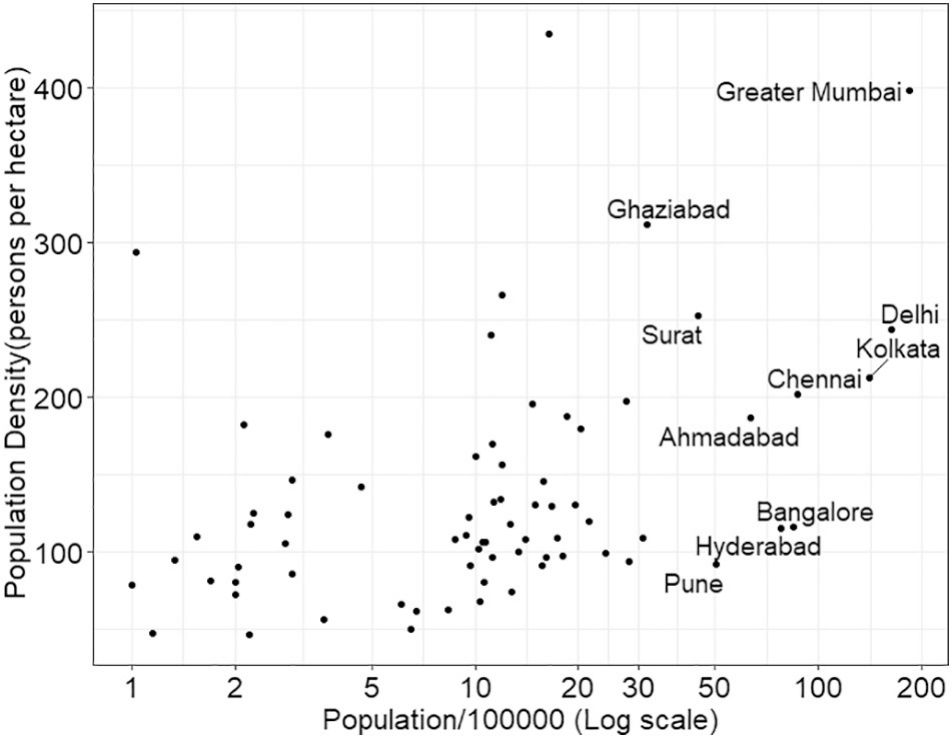
Population size and density

Fig. 2 shows scatterplot of population density and the log transformed population of the cities, indicating a linear relationship between the two. In all the scatterplots presented in this study, we have indicated the names of only the ten most populated cities, due to space constraints. The Pearson correlation between the two variables is 0.4 and is significant at 99% confidence interval (Table 2). Clearly, as the population of the cities increase, so does their density. It shows that association of travel pattern with the population density should also take population into account. However, it is important to note that there is a wide scatter at all population levels, and as we will see, this is true for all the relationships we examine in this paper. Since the population is log transformed, the actual relationship between the two variables is non-linear.

### 3.4 Population density and share of long trips

**Fig. 3:**
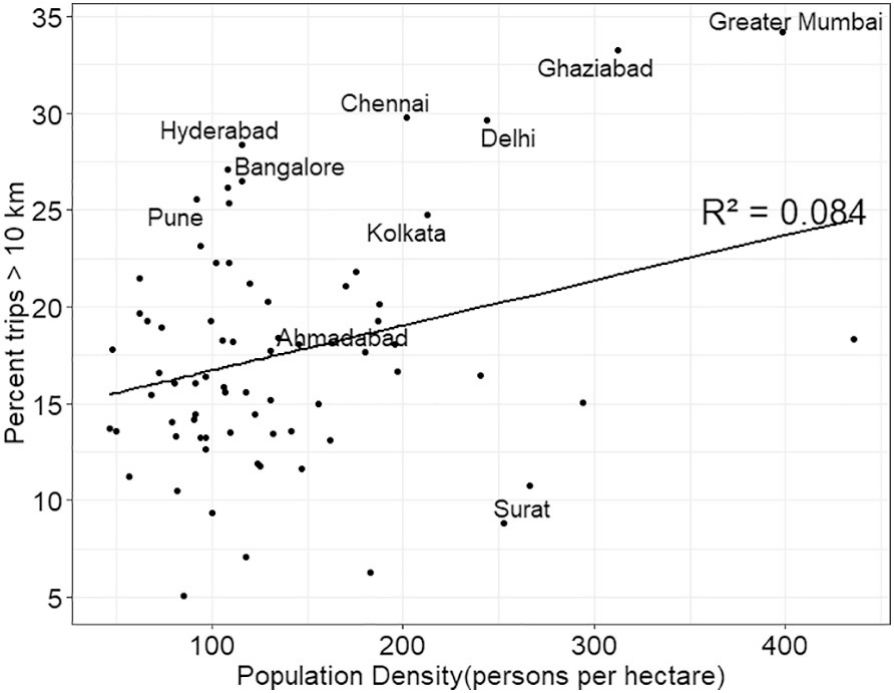
Share of long trips with respect to population density.

Fig. 3 presents the share of long trips (longer than 10 km) with respect to the population density. There is a large variation of long trips for a given level of density. Interestingly, the share of long trips has a positive association with the population density. This is counterintuitive as higher density levels are believed to facilitate smaller travel distances. We computed a beta regression of the share of long trips with population density, natural log of population, and car ownership as explanatory variables. Population and car ownership control for the size and the income levels, respectively. The regression shows a significant positive relationship with the population and the car ownership, and an insignificant relationship with population density (see Appendix A). This means that the slope shown in Fig. 3 is an indicator of density's relationship with the population, while density itself has no independent effect on travel distance after controlling for the population.

### 3.5 Size of the city and share of long trips

**Fig. 4:**
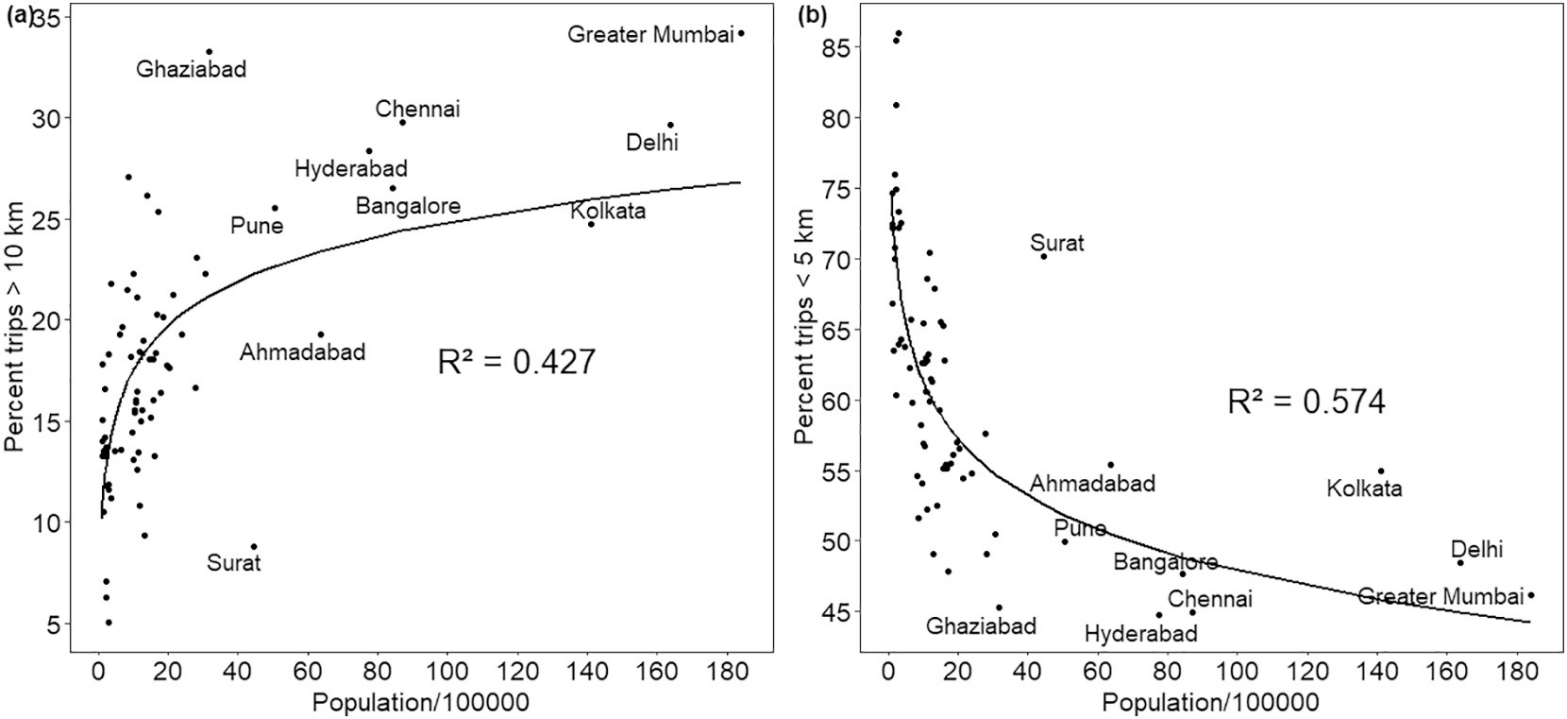
Trip distance distribution with city population.

**Fig. 5:**
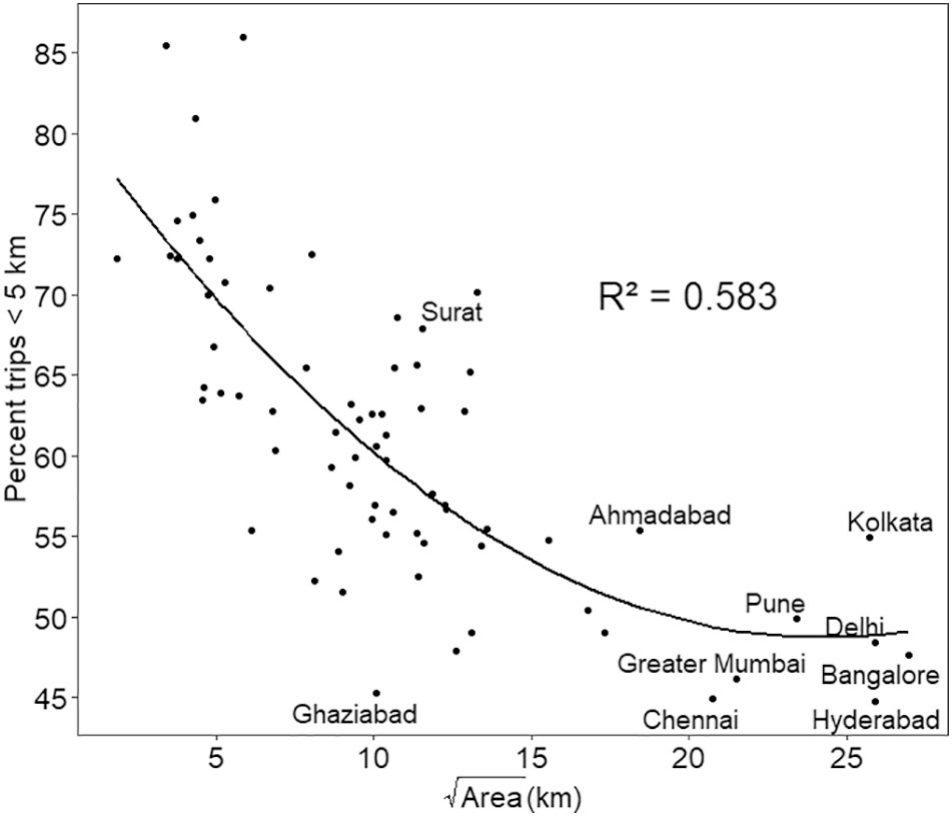
Share of short trips with respect to size of the city

Fig. 4 presents the relationship between the travel distance and the population, for trips longer than 10 km and those shorter than 5 km. While the former represents trips that are more likely to be travelled using motorised modes, the latter represent those which are more likely to be travelled using non-motorised modes. The data show that while small cities have shorter travel distances, there is a great amount of scatter among cities with a similar population, except those with population > 2 million. Both graphs show that beyond the population size of 2 million, travel distance is largely independent of the population. Fig. 5 shows the relationship between the share of short trips (<5 km) and the square root of the size of the cities. The square root converts the size from area unit to distance unit and can be hypothesized to represent the size of the city in terms of distance. It can be seen that beyond 12 km (or 145 km^2^), the share of short trips remains almost constant, though there are a few much smaller cities with share of short trips similar to that of the large cities.

### 3.6 Population density and travel mode shares

**Fig. 6:**
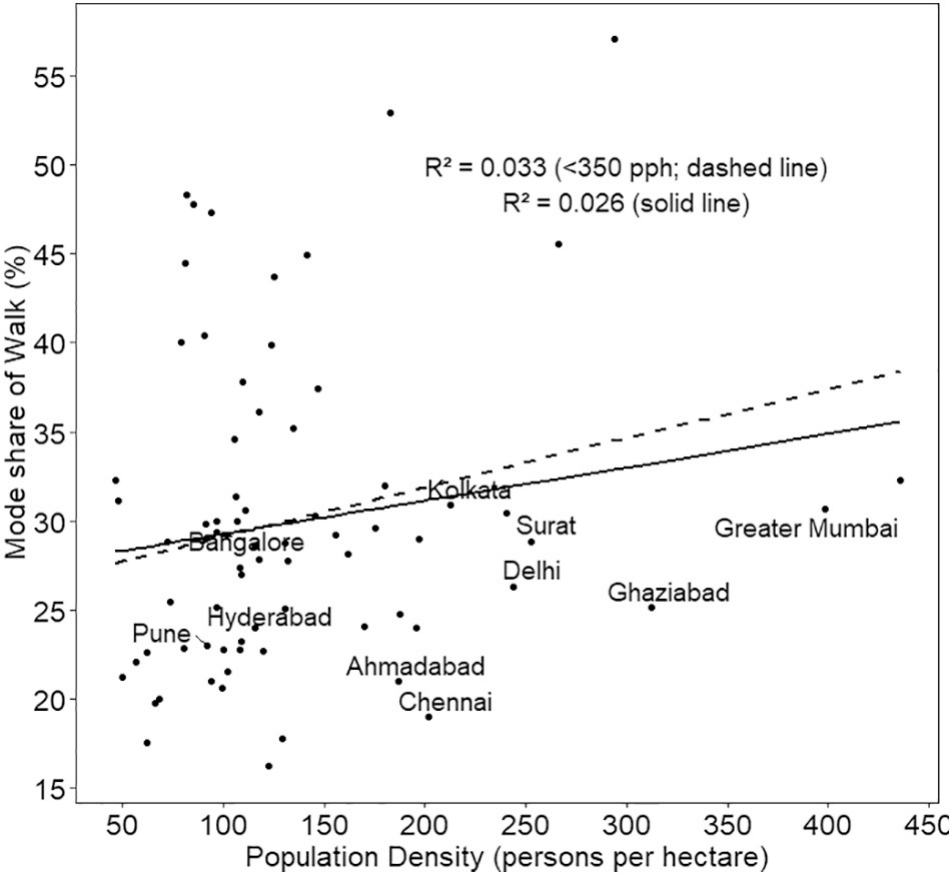
Mode share of walking with respect to population density.

**Fig. 7:**
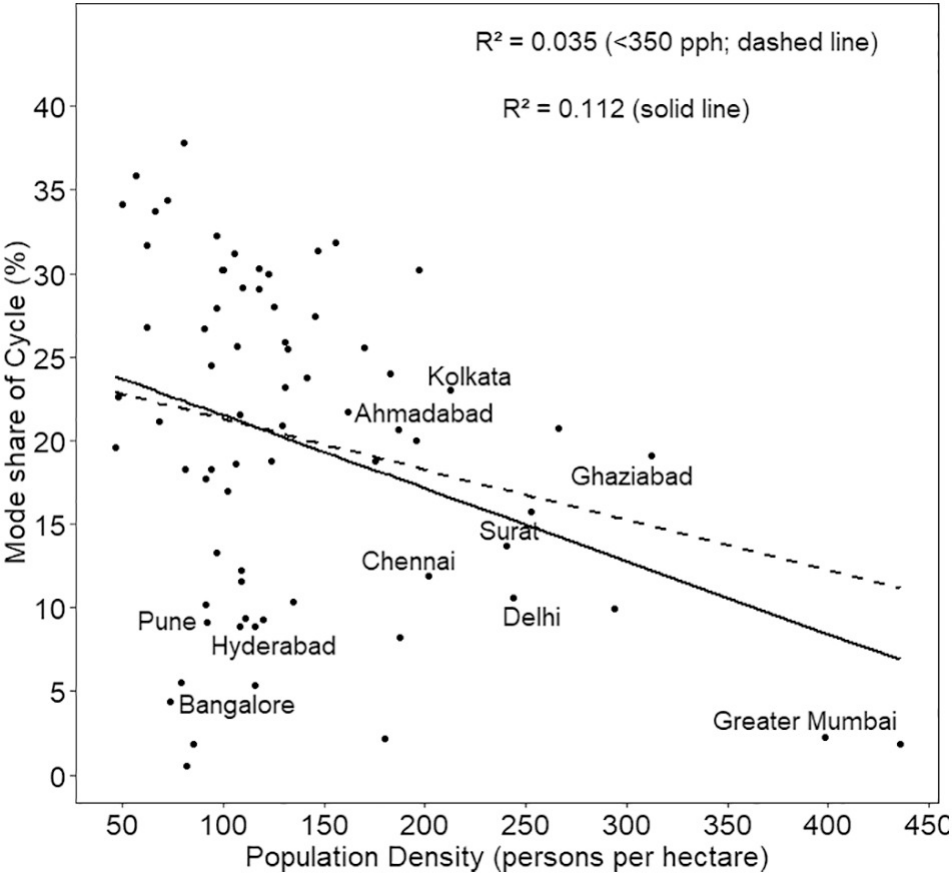
Mode share of cycle with respect to population density.

**Fig. 8:**
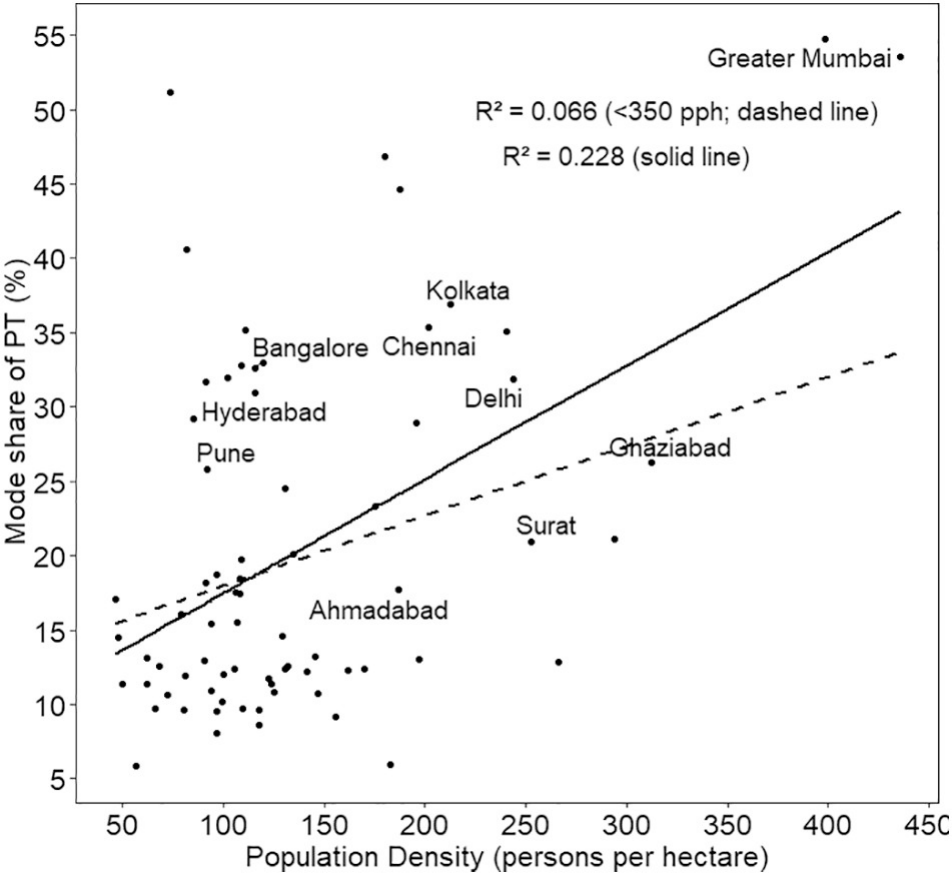
Mode share of PT with respect to population density.

**Fig. 9:**
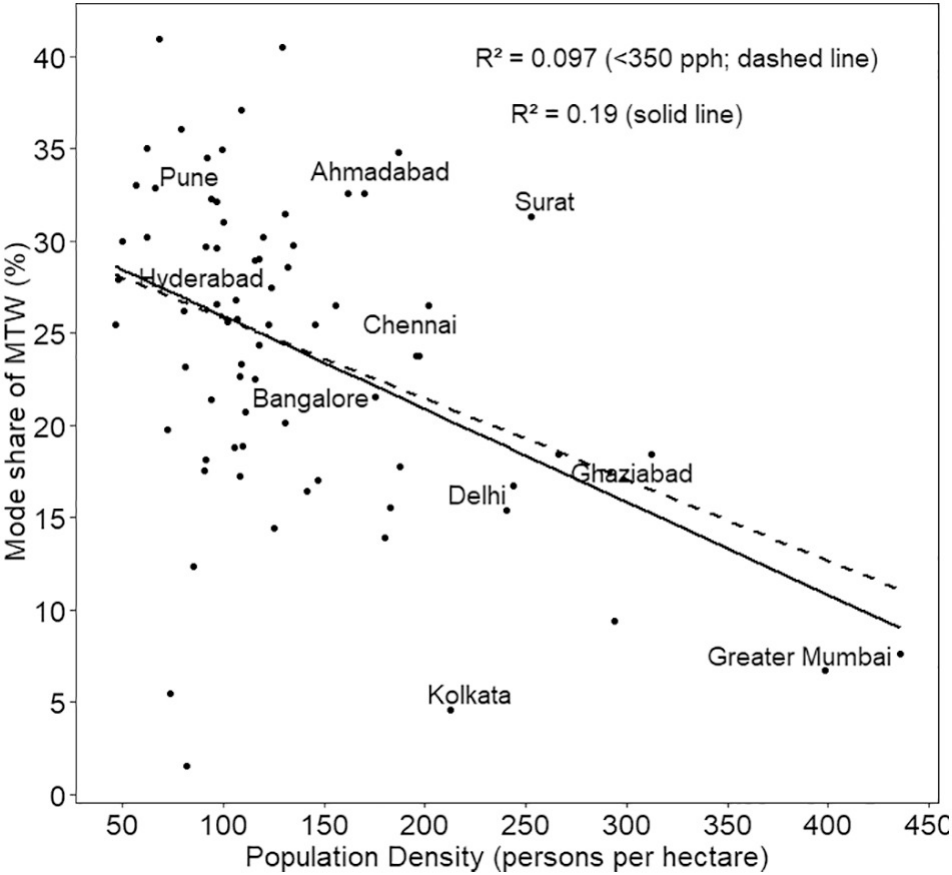
Mode share of MTW and population density.

**Fig. 10:**
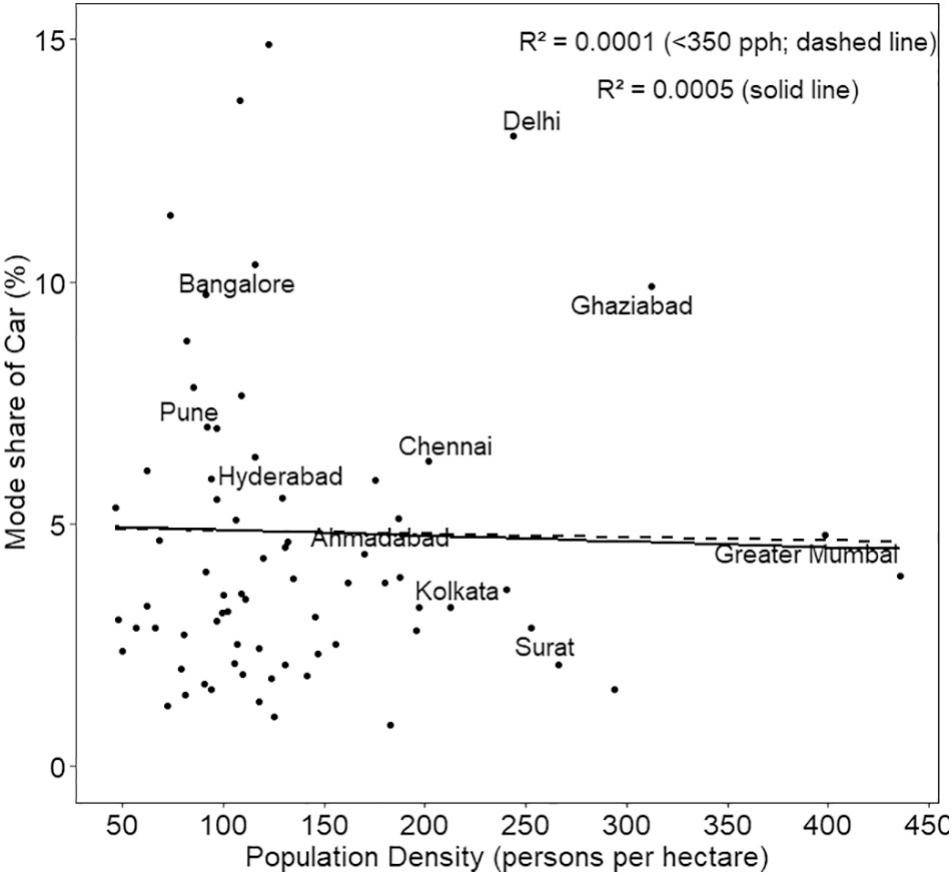
Mode share of car with respect to population density (excludes Gurgaon city with 28% share of car trips and density of 108 pph, for better representation of the plot).

The associations of the shares of five travel modes with density are also shown as scatter plots in Fig. 6, Fig. 7, Fig. 8, Fig. 9, Fig. 10, with respective R^2^ values for a linear relationship. What is noteworthy is the very wide scatter of mode shares at all population density levels except for the cities above 250–300 pph, where the number of cities itself is limited. For instance, for the density of 120–125 pph, walk share varies from 16% to 44%, cycle from 9% to 21%, PT from 9% to 33%, MTW from 14% to 40%, and car from 1% to 15%. In case of walk and car, almost no variance is explained by the density.

In the scatter plots, the two cities with the highest population density— Greater Mumbai and Kannur—, are outliers with population density higher than 350 pph. To assess the sensitivity of our results to these two cities, we estimated R^2^ for the set of cities excluding the outliers, referred to as <350 pph in the plots. The resulting linear plots and corresponding R^2^ values have been shown in the same plots for all the five travel modes (Fig. 6 through Fig. 10). R^2^ has reduced significantly for cycle, PT, MTW as well as car by 50% to 80%, with an exception for walk in which case R^2^ value increased.

Using beta regression, we investigate the relationship of population density with mode share (expressed as a fraction) of walk, cycle, PT, MTW, and car. In order to assess an independent effect of the density, we included four explanatory variables— population of the city (transformed using natural log), and ownerships of cycle, MTW, and car. The correlation of GDP per capita is much higher with car ownership than with 2 W ownership (0.77 and 0.50, respectively at 99% CI; see Table 2). In addition, car ownership has strong correlation with population (log transformed—0.48 as well as absolute—0.3), size of the city, and share of trips between 6 km and 20 km (>0.50). Therefore, coefficient of car ownership should be interpreted as not only vehicle ownership but also as a proxy for city's income, its size and longer travel distance.

Table 3 presents regression results for the mode share of the five travel modes. Coefficients with statistical significance at 90% CI or more are shown in bold. For each of the models, we also carried out stepwise regression, starting with only population density as the independent variable and then sequentially adding other variables. There is no indication that the effect of population density is affected with the inclusion of other variables.

**Table 3:** Beta regression models of mode-specific commute shares (coefficients with standard error in parentheses).

	Walk	Cycle	PT	MTW	Car
Intercept	**1.943 (0.431)**	**−2.216 (0.763)**	**−4.451 (0.664)**	−0.735 (0.509)	**−4.807 (0.441)**
Ln (population)	**−0.183 (0.037)**	−0.009 (0.064)	**0.291 (0.056)**	**−0.075 (0.043)**	**0.092 (0.036)**
Population density	**0.002 (0.0004)**	−0.001 (0.0009)	0.0009 (0.0007)	**−0.002 (0.001)**	**−0.001 (0.0004)**
2W ownership	**−0.693 (0.386)**	0.666 (0.675)	**−1.976 (0.591)**	**4.530 (0.434)**	**−0.714 (0.372)**
Car ownership	−0.558 (0.699)	**−3.638 (1.344)**	**2.638 (1.052)**	**−5.863 (0.793)**	**9.980 (0.483)**
Cycle ownership	**−0.594 (0.182)**	**2.519 (0.309)**	**−1.804 (0.311)**	0.102 (0.190)	−0.107 (0.188)
Pseudo R^2^	0.625	0.678	0.622	0.741	0.854

Coefficients with statistical significance at 90% CI or more are shown in bold.

Final model presented in Table 3 shows that population density has a positive association with walk and PT, while a negative relationship with cycle, MTW and car. However, the association is statistically significant only for walk, MTW and car. The magnitude of coefficient is of the order of 10^−4^. This implies that even a 50 pph increase of density will result in almost no change in the mode share of car, and less than a unit percent change in the other four travel modes. We also developed the regression models to the subset of dataset excluding cities with population > 350 pph. We found that for all the modes, the sign as well as order of magnitude remained the same, except PT, in which case, coefficient reduced by an order of magnitude from 1.4 × 10^−4^ to 4.1 × 10^−5^. Thus the removal of outliers from the dataset has reduced the strength of relationship between density and PT usage even more.

Population has a statistically significant coefficient in case of walk, PT and car— negative for walk and positive for the other two. The coefficients imply that an increase in the population, for instance, from 1 million to 2 million, will result in 4% increase in the PT share and a similar decrease in the walking share. Larger cities are more likely to have higher usage of PT and car, and lower share of walking. Also, city-based government-run bus services are available in only a handful of large cities of India—Mumbai, Delhi, Kolkata, Chennai, Bangalore, Hyderabad, Ahmedabad, Pune, Chandigarh, Thane, Navi Mumbai, Kolhapur, and Solapur (CIRT, 2011). The bus services in the rest of the cities are either provided by state-level government-run services (mostly for inter-city or for few major intra-city routes) or intra-city private operators.

Car ownership has a negative coefficient in case of walk, cycle, and MTW, which is also expected, however, it has a positive coefficient in case of PT mode share, which is counterintuitive. However, this can be explained if the car ownership is considered as a proxy of other city-level covariates, such as population, size, and long trips, as discussed above. Walk, cycle and MTW are associated with shorter travel distance (< 5 km), while PT with longer travel distance (see Table 2). Thus cities with higher car ownership and, therefore, with longer travel distance, are more likely to have higher share of PT than the other three modes.

**Fig. 11:**
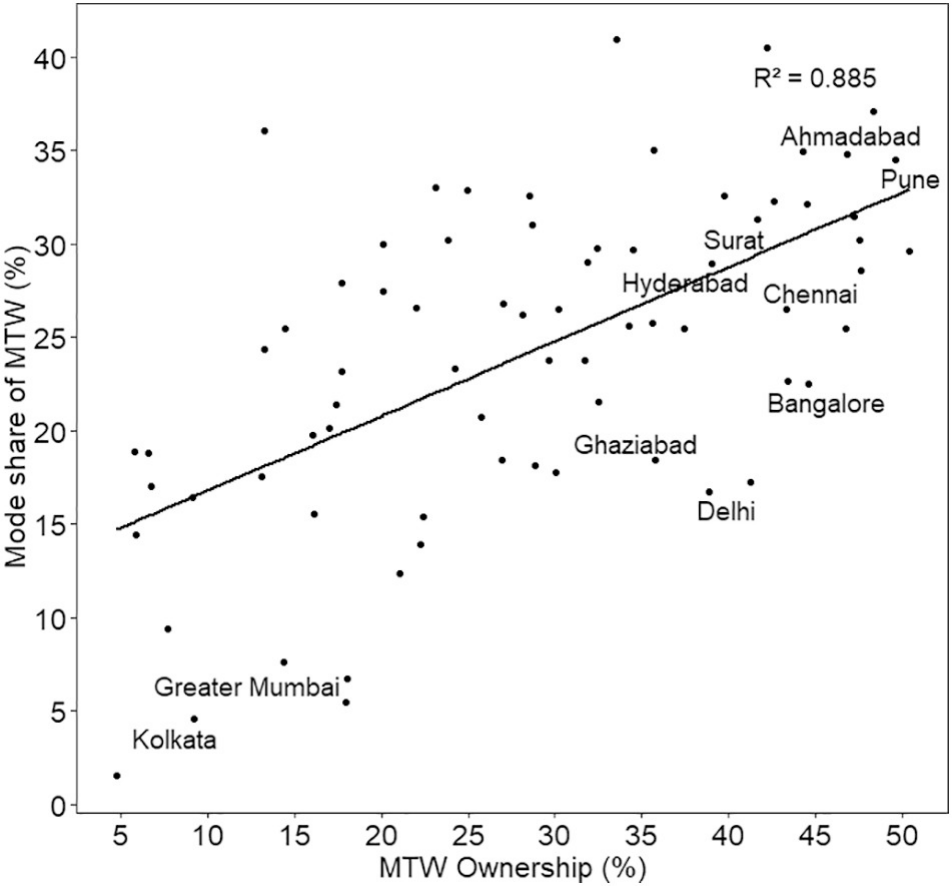
Mode share of MTW with respect to its ownership.

**Fig. 12:**
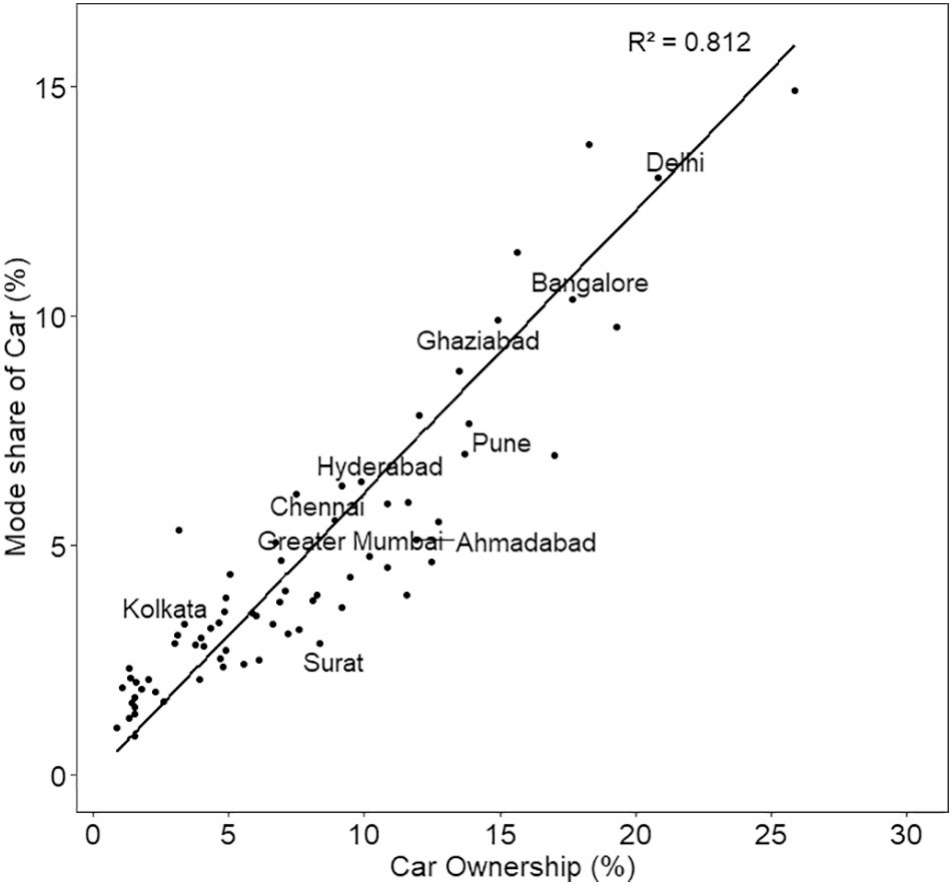
Mode share of car with respect to its ownership.

The coefficient of population density for MTW share is an order of magnitude higher than the coefficient for car share. To understand the difference in the usage of two modes, we present the scatter plots of their mode shares with respect to their corresponding ownership levels (Fig. 11 and Fig. 12). The starkest difference between the two plots is the scatter, which is much higher in the case of MTW. In other words, the variation of car use for a given level of car ownership is much less, compared to that of MTW. On an average, in a city, there are almost four MTW owners for every one car owner (Table 1). Therefore, with high levels of MTW ownership, there is much higher variation in how households choose to use this mode based on factors such as demography, built environment, and availability of public transit. In contrast, car use appears to be strongly associated with the ownership. It can be seen in Table 2 that mode share of MTW has a statistically significant negative correlation with PT ownership (−0.44 at 99% CI), while mode share of car has a weak positive correlation. Therefore, for a given level of MTW ownership, its use is likely to vary by availability of PT, as MTW owners are also likely to use PT while car owners are less likely to do so.

One could argue that the population density may affect mode share through vehicle ownership. Therefore, inclusion of vehicle ownership categories as the covariates may reduce the independent effect of density on mode share. In order to test this hypothesis, we carried out regression of the mode share of five modes, and excluded ownership of vehicles as explanatory variables. In the absence of the vehicle ownership, to control for income level of the city, we added GDP per capita as one of the covariates. This regression was carried out for 66 cities for which GDP data is available. In all the modes, the sign as well as the order of magnitude of the coefficient remained the same (10^−4^ or less), while the statistical significance changed for some (see Appendix A). With respect to the population density, the association with walk and MTW remained the same, with car became insignificant, and with PT and cycle became significant. Therefore, the conclusion regarding the effect of density is not sensitive to the type of explanatory variables included.

### 3.7 International perspective

**Fig. 13:**
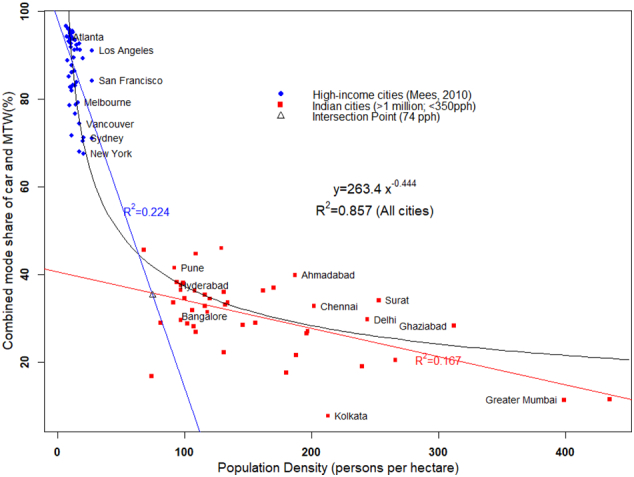
Mode share of private motorised modes and population density using data from Mees (2010) and cities from this study with population 1 million or higher.

The analyses in the previous sections aimed at understanding variation of travel behaviour with respect to city-level correlates for cities within India. In order to put the set of Indian cities discussed above in an international perspective, we employed the dataset of high-income cities reported by Mees (2010). For his analysis, Mees focused on 50 cities in the US, Canada and Australia. Mees improved the density estimates reported by Newman and Kenworthy (1999) (see Fig. 1) for the Canadian and the Australian cities using a definition of built-up area consistent with that of the cities in the US. The dataset provides population, overall urban density, and mode share for journey to work classified as car, public transport, walking, cycling and other. Fig. 13 presents a scatter plot of the set of cities reported by Mees (2010) and 42 Indian cities from this study with the population of 1 million or more. For the Indian cities, we excluded two outlier cities with density higher than 350 pph, and included MTW along with the share of car, as the two modes together represent the share of private motorised modes. The R^2^ is 0.85 for a power relationship across all the 92 cities.

#### 3.7.1 A bi-linear form?

An alternative way to look at the power relationship shown in Fig. 13 is to consider it as a combination of two independent linear relationships, i.e. a bi-linear form. According to this, the relationship for low-density, high-income cities is governed by a different mechanism than the relationship for high-density, Indian cities. Fig. 13 also shows the two linear relationships—one using the set of high-income cities reported in Mees (2010) and the other using the set of Indian cities. The former has a steep slope with increase in the density accompanied by a rapid reduction of private vehicle use, while the latter has a much flatter slope, indicating minimal impact of density on private vehicle use.

## 4 Discussion

In this study, we found that the direction of association between the density and the mode of travel is mostly as expected—positive with walking and PT, and negative with cycle, MTW and car. The negative association with the cycling is counterintuitive—higher density should facilitate travel distance convenient for cycling. The regression accounted for other city-level correlates such as the population and the income level. The magnitude of association indicates that there is no evidence that an achievable change in the overall urban density results in any significant change in the shares of any mode. Even an increase of 50 pph in the density will only result in a sub-unit change in the percent shares of travel modes.

We found that a more important factor affecting the travel patterns across the cities is the population. As cities become larger in population and size (area), the travel distance increases, and the trips travelled by walk, cycle and MTW reduce and those by car, bus, and train increase. We found that each travel mode is associated with a specific range of travel distance— car, bus and train with long trips (>10 km), and walk, cycle and MTW with short trips (<10 km). Therefore, an increase in overall PT share as cities become larger (or more populated) is because of a need to travel in motorised modes as trips become longer. Since population and density are positively correlated, increase in PT share is also positively correlated with density. Therefore, density in this case seems a facilitator for a feasible public transport system, rather than the cause for higher PT usage.

A major part of the discourse relating density and travel behaviour has been contributed through an international perspective rather than within-country differences among the cities, and the former has been pioneered by Newman and Kenworthy, 1989, Newman and Kenworthy, 1999. The argument linking density with per capita transport energy usage as put forth by Newman and Kenworthy has been criticised by Mindali et al. (2004) and Mees (2010). Mindali et al. carried out multivariate analysis and found that, among the North American, Australian, and European cities, overall urban density did not show any correlation with transport energy use. The authors pointed out in the graph presented by Newman and Kenworthy (1989) for 1980 data that, with similar level of population density (<25 pph), Australian cities consume half as much gasoline per person as their American counterparts.

Similar to Mindali et al. (2004), using the trend line published in Newman and Kenworthy (1999) for 1990 data, Mees (2010) pointed out that the relationship ignores the high variation of transport energy usage for a given level of density. Mees also added that the density estimates used by Newman and Kenworthy (1999) for the European and the Canadian cities were overestimated. Thus, correcting for the overestimation, European and Canadian cities will be shifted on the left along x-axis, closer to the American cities. This would further imply that all American, Canadian, and European cities within a small range of population density have a large variation of transport energy use ranging from 15,000 M joules (MJ) per capita to 50,000 MJ. This again shows that travel behaviour is relatively independent of density.

Mees (2010) used the revised density estimates for Australian, American, and Canadian cities and presented scatterplot of density with commute mode share of car, PT, walk, and a combination of PT and walk. Using the best-fitted curve, Mees pointed out that the R^2^ values are much lower (<0.3) than those reported by Newman and Kenworthy (1999) (0.8–0.9), and concluded that density is indeed not relevant.

The conclusion made by Mees (2010) may be correct about the cities in the three countries, however, their claim that it was revealed because of better estimates of density may be wrong. We estimated R^2^ of 0.22, using the best fit for the data of Australian, American, and Canadian cities reported by Mees (2010) (see Fig. 13). When we plot this data along with Indian cities, R^2^ of best fit increases to 0.85 (Fig. 13). Therefore, the same set of data points along with those of Indian cities exhibit a strength of relationship which is completely in disagreement with what Mees (2010) tried to explain. Moreover, the trend looks similar to that of Newman and Kenworthy (1999).

Therefore, the question is not whether a power relationship, such as the one shown in Fig. 13, holds for cities across the world with a vast range of population density. Instead, the question is how to interpret such a relationship, which lends us to the classic debate of correlation and causality. Since, density is related to multiple factors, therefore, whether density affects travel behaviour directly or through other variables with which it is correlated has also been questioned by researchers previously (Ewing & Cervero, 2001).

It has been argued that it is possibly other factors, such as traffic congestion, which go along with higher density that may be responsible for its linkage with travel behaviour (Handy, 1995; Steiner, 1994; Miller & Ibrahim, 1998). Newman and Kenworthy (2006) presented a relationship between density and transit use for planning zones in the Los Angeles region, with the former explaining 96% of the variation in transit use. Modarres (2013) criticised this relationship based on the correlation between density and income level. Modarres argued that in the case of Los Angeles, commuters living in high-density neighbourhoods are more likely to use public transport but they are also as likely to be low-income minorities and immigrants. The author concluded that “space and the built environment matter, but who lives where is equally and, in some cases, more important”.

The above argument based on the correlation between density and income level (that is, denser neighbourhoods tend to be low-income) can also be used to explain the differences among countries. One of the major problems with the power function presented in Fig. 13 is that it ignores the vast gap between the income levels of the cities in the two clusters which differs by an order of magnitude. This wrongly highlights density as a strong determinant of travel behaviour. Among the cluster of the high-income cities, US had an average per capita income (in US dollars) of $50,000 in 2011, Canada, $52,000 and Australia, $62,000, while low and middle income countries are less than $ 15,000. Therefore, high density of Indian cities and higher share of non-motorised and public transport modes is less likely a result of design and policy than a market outcome in a setting with low-income level and low vehicle ownership. A bi-linear relationship defining the two clusters under their individual lines is a better representation than a single power relationship across the two clusters. The former is an explicit representation of two different mechanisms under play—one which is applicable to the high-income, heavily motorised countries, and other to the low-income countries.

## 5 Conclusions

We found no evidence to suggest that an increase in overall urban density has any effect on travel behaviour in cities in India, after accounting for population and income level. It appears that once density levels are greater than ~80 persons per hectare, car use is likely to be low and other factors determine travel mode choices than density. Our findings have significant implications in terms of land-use and transport policies in most cities around the world. The further densification of cities with >80 pph is not likely to result in better usage of public transportation modes. Given the large variation of travel behaviour for a given level of population density, future research should focus on understanding details of street and urban layout that result in large variations in travel behaviour. More detailed analysis will be feasible as Census publishes data at city or ward level in the near future. A major limitation of this study is that the conclusions are based on city-level aggregate variables, such as overall density as well as commute travel pattern.

## Figures and Tables

**Table A1:** Linear regression of share of long trips(>10 km)^a^.

	Coeff.
Intercept	−3.754 (0.399)
Ln (Population)	0.234 (0.032)
Population Density	−0.0006 (0.0005)
Car ownership	1.478 (0.574)
Pseudo R^2^	0.59

a Share of long trips expressed as fraction.

**Table A2:** Beta regression of mode shares^a^ including GDP per capita as an income proxy.

	Walk	Cycle	PT	MTW	Car
Intercept	2.356 (0.42)	0.552 (0.902)	−4.971 (0.775)	−2.174 (0.658)	−8.6 (0.811)
Ln (Population)	−0.216 (0.037)	0.162 (0.081)	0.091 (0.064)	0.177 (0.057)	0.052 (0.061)
Population Density	0.002 (0.0004)	−0.004 (0.001)	0.004 (0.001)	−0.005 (0.001)	−0.0008 (0.001)
Ln (GDP per capita)	−0.082 (0.060)	−0.561 (0.133)	0.265 (0.108)	−0.119 (0.09)	0.73 (0.097)
Log likelihood	95.2	66.32	77.24	78.26	164.8
Pseudo R^2^	0.49	0.266	0.437	0.308	0.506

a Mode shares expressed as fractions.

**Table A3:** Regression model for mode share of walking as dependent variable.

	Model 1	Model 2	Model 3	Model 4	Final model
Intercept	−0.966 (0.096)	2.399 (0.391)	2.652 (0.364)	2.356 (0.383)	1.943 (0.431)
Ln (Population)		−0.261 (0.03)	−0.255 (0.027)	−0.222 (0.031)	−0.183 (0.037)
Population density	0.001 (0.001)	0.003 (0.0005)	0.002 (0.0004)	0.002 (0.0004)	0.002 (0.0005)
Cycle ownership			−0.634 (0.172)	−0.713 (0.173)	−0.594 (0.182)
Car ownership				−1.196 (0.609)	−0.559 (0.699)
MTW ownership					−0.693 (0.386)
Log likelihood	77.66	102.8	109	110.9	112.4
Pseudo R^2^	0.02816	0.5034	0.5812	0.6059	0.625

**Table A4:** Regression model for mode share of cycling as dependent variable.

	Model 1	Model 2	Model 3	Model 4	Final model
Intercept	−1.067 (0.167)	−0.821 (0.921)	−2.014 (0.656)	−2.583 (0.678)	−2.216 (0.764)
Ln (Population)		−0.019 (0.07)	−0.044 (0.048)	0.026 (0.054)	−0.009 (0.064)
Population density	−0.003 (0.001)	−0.002 (0.001)	−0.001 (0.001)	−0.001 (0.001)	−0.001 (0.001)
Cycle ownership			2.867 (0.303)	2.626 (0.297)	2.519 (0.309)
Car ownership				−2.88 (1.077)	−3.638 (1.345)
MTW ownership					0.666 (0.675)
Log likelihood	64.12	64.16	99.33	102.6	103.1
Pseudo R^2^	0.09629	0.09715	0.6325	0.6661	0.6775

**Table A5:** Regression model for mode share of public transport as dependent variable.

	Model 1	Model 2	Model 3	Model 4	Final model
Intercept	−1.927 (0.143)	−4.348 (0.769)	−3.613 (0.603)	−3.404 (0.635)	−4.451 (0.664)
Ln (Population)		0.184 (0.058)	0.211 (0.045)	0.188 (0.051)	0.291 (0.056)
Population density	0.004 (0.001)	0.003 (0.001)	0.002 (0.001)	0.002 (0.001)	0.001 (0.001)
Cycle ownership			−2.203 (0.301)	−2.154 (0.308)	−1.804 (0.311)
Car ownership				0.824 (0.97)	2.638 (1.052)
MTW ownership					−1.976 (0.591)
Log likelihood	70.83	75.53	94.32	94.69	99.74
Pseudo R^2^	0.2013	0.2989	0.5272	0.5774	0.6227

**Table A6:** Regression model for mode share of MTW as dependent variable.

	Model 1	Model 2	Model 3	Model 4	Final model
Intercept	−0.791 (0.125)	−2.531 (0.669)	−2.979 (0.644)	−3.147 (0.684)	−0.735 (0.509)
Ln (Population)		0.136 (0.051)	0.13 (0.048)	0.152 (0.055)	−0.075 (0.043)
Population density	−0.003 (0.001)	−0.004 (0.001)	−0.003 (0.001)	−0.004 (0.001)	−0.002 (0.001)
Cycle ownership			1.028 (0.29)	0.931 (0.297)	0.102 (0.191)
Car ownership				−0.937 (1.02)	−5.863 (0.793)
MTW ownership					4.53 (0.439)
Log likelihood	73.23	76.87	83.33	83.78	122
Pseudo R^2^	0.1486	0.2141	0.3271	0.3383	0.7408

**Table A7:** Regression model for mode share of car as dependent variable.

	Model 1	Model 2	Model 3	Model 4	Final model
Intercept	−2.977 (0.159)	−6.483 (0.828)	−6.093 (0.821)	−4.334 (0.381)	−4.807 (0.441)
Ln (Population)		0.27 (0.062)	0.271 (0.06)	0.049 (0.029)	0.092 (0.036)
Population density	0 (0.001)	−0.002 (0.001)	−0.002 (0.001)	−0.001 (0.0005)	−0.001 (0.0005)
Cycle ownership			−0.765 (0.379)	−0.281 (0.169)	−0.107 (0.188)
Car ownership				9.438 (0.399)	9.98 (0.483)
MTW ownership					−0.714 (0.372)
Log likelihood	155.9	164.7	166.5	230.6	232.4
Pseudo R^2^	0.0000209	0.2864	0.3268	0.8468	0.8539

## Appendix B Supplementary data

Supplementary material Image 1

## Footnotes

1 1 US dollar = ~70 Indian Rupees, in 2019.
